# Production and characterization of amplified tumor-derived cRNA libraries to be used as vaccines against metastatic melanomas

**DOI:** 10.1186/1479-0556-3-6

**Published:** 2005-08-22

**Authors:** Jean-Philippe Carralot, Benjamin Weide, Oliver Schoor, Jochen Probst, Birgit Scheel, Regina Teufel, Ingmar Hoerr, Claus Garbe, Hans-Georg Rammensee, Steve Pascolo

**Affiliations:** 1 CureVac, Paul Ehrlich Strasse 15, 72076 Tübingen, Germany; 2 University of Tübingen, Institute for Cell Biology, Department of Immunology; Auf der Morgenstelle 15; 72076 Tübingen, Germany; 3 Section for Dermatological Oncology, Tübingen University Hospital, Liebermeisterstraße 25, 72076 Tübingen, Germany

## Abstract

**Background:**

Anti-tumor vaccines targeting the entire tumor antigen repertoire represent an attractive immunotherapeutic approach. In the context of a phase I/II clinical trial, we vaccinated metastatic melanoma patients with autologous amplified tumor mRNA. In order to provide the large quantities of mRNA needed for each patient, the Stratagene Creator™ SMART™ cDNA library construction method was modified and applied to produce libraries derived from the tumors of 15 patients. The quality of those mRNA library vaccines was evaluated through sequencing and microarray analysis.

**Results:**

Random analysis of bacterial clones of the library showed a rate of 95% of recombinant plasmids among which a minimum of 51% of the clones contained a full-Open Reading Frame. In addition, despite a biased amplification toward small abundant transcripts compared to large rare fragments, we could document a relatively conserved gene expression profile between the total RNA of the tumor of origin and the corresponding *in vitro *transcribed complementary RNA (cRNA). Finally, listing the 30 most abundant transcripts of patient MEL02's library, a large number of tumor associated antigens (TAAs) either patient specific or shared by several melanomas were found.

**Conclusion:**

Our results show that unlimited amounts of cRNA representing tumor's transcriptome could be obtained and that this cRNA was a reliable source of a large variety of tumor antigens.

## Background

The identification by van der Bruggen *et al*. [1] of the first tumor associated (TAA) antigen recognized by specific cytotoxic T lymphocytes (CTLs) in melanoma patients boosted the development of anti-cancer immunotherapy strategies. During the last years, vaccination protocols targeting differentiation antigens (MART-1/Melan-A [2,3], gp100 [4], Tyrosinase [5,6]) or cancer-testis antigens (MAGE [1,7], NY-ESO1 [8]) were tested and showed encouraging results [9-11].

However, a growing body of evidence suggests that, instead of using defined antigens, targeting the whole spectrum of tumor antigens would represent an alternative, potentially more efficacious method [12-14]. Indeed, the use of total tumor material for vaccination allows the development of B and T cells directed against a large variety of known but also unknown TAAs [15]. In addition, stimulating such a large spectrum of specific effectors directed against multiple epitopes restricted by diverse HLA class I and II types would reduce the risk of tumor escape through antigen loss or MHC downregulation [16-19]. Finally, another advantage of the whole tumor approach is that, in an autologous setting, patient's TAAs eventually stemming from tumor-specific somatic mutations could be targeted [20,21].

In order to vaccinate patients with the whole spectrum of TAAs, several methods were developed. In 1998, Soiffer *et al*. [22] disclosed the results obtained by vaccinating patients with autologous irradiated tumor cells engineered to produce GM-CSF. The same year, Nestle *et al*. [23] showed partial or complete tumor remissions in six melanoma patients vaccinated with dendritic cells (DC) loaded with autologous tumor lysate. Alternatively, Boczkowski *et al*. [24] reported that mouse DCs pulsed *in vitro *with tumor RNA could trigger an anti-tumor immunity *in vivo*. Several groups further developed and optimized those different strategies [25-27] but faced the limitation imposed by the requirement of large amounts of tumor tissue for lysate preparation or for sufficient RNA yields extraction. In order to overcome this drawback, Boczkowski *et al*. [28] modified the SMART method (BD Biosciences Clontech, Palo Alto, CA) in order to *in vitro *transcribe tumor cDNA and performed therefore a one-step amplification of tumor mRNA. Transfected into antigen presenting cells (APCs), this amplified cRNA was shown *in vitro *to induce anti tumor immunity [29,30]. As an alternative vaccination method, Hoerr *et al*. [31] demonstrated the capacity of mRNA coding for defined antigens or of total cRNA to trigger an antigen-specific immune response after direct intra-dermal injections of the ribonucleic acid. Similarly, Granstein *et al*. [15] showed protection against S1509 tumor cells in mice that received three intradermal injections of total RNA extracted from S1509 cells. Although still marginally studied compared to mRNA-loaded DC vaccines, the direct injection of mRNA represents a technology that offers the important advantage to circumvent the time and money consuming steps of generation of DCs.

In 2003, we initiated the first phase I/II clinical study to test the feasibility, safety, and efficacy of a vaccine composed of autologous amplified tumor mRNA in stage III/IV patients with metastatic melanoma (The detailed evaluation of the toxicity, clinical and immunological efficacy of this treatment will be reported in a following manuscript). Fifteen patients received from 3 to 16 intradermal injections of 200 μg of amplified autologous tumor cRNA. The amount of injected RNA was limited by the maximal intradermal injection volume (100 μl) and set according to the preclinical results which indicated that a concentration of ca. 0.8 μg/μl was leading to a good gene transfer. The injection's schedule consisted in applications every two weeks of four injections and then once every month. It was decided empirically since no previous data on the toxicity and efficacy of this immunization method are available in humans. In mice one injection was shown to be sufficient to trigger an immune response [31]. However, in cancer patients, a sustained stimulation of the immunity is probably requested in order to get an efficient anti-tumor immune response. According to this protocol, the required amount of cRNA for a complete therapy was between 0.6 and 3.2 mg per patient (table 1). In order to get unlimited amounts of product, a new method for the amplification of the tumor mRNA was developed. Briefly, a cDNA library was generated from tumor RNA using the SMART (Switch Mechanism At the 5'end of RNA Templates) system (BD Clontech) and then was cloned in our RNActive™ vector (CureVac GmbH), amplified in *Escherichia coli*, and finally transcribed *in vitro*. As opposed to the protocol described by Boczkowski *et al*. [28] in which the PCR-amplified tumor cDNA library was directly used as template for the *in vitro *transcription (resulting in limited cRNA amounts), the method applied in our laboratory provided us with unlimited amounts of the tumor-derived cRNA.

**Table 1:** Summary of mRNA libraries and clone analysis. In the case of MEL14, total RNA was extracted from ~5 × 10^4 ^pleural tumor cells (NA: Not applicable)

**Patients**		**Weight of tumor sample (mg)**	**Quantity of extracted total RNA (μg)**	**Number of clones (cfu)**	**Size range of analyzed clones (nt)**	**Quantity of mRNA library prepared (mg)**	**Number of injection performed**
**1**	**MEL01**	32.4	9.4	1 × 10^5^	500 – 4000	2.8	10
**2**	**MEL02**	33	10.5	1 × 10^5^	200 – 8000	5.0	12
**3**	**MEL03**	33.6	26.3	5 × 10^5^	250 – 1000	1.9	6
**4**	**MEL04**	38	36.7	2 × 10^5^	400 – 3500	3.6	8
**5**	**MEL05**	85	14.2	2 × 10^5^	500 – 1000	5.0	13
**6**	**MEL06**	60	115.2	3 × 10^5^	300 – 1200	4.0	16
**7**	**MEL07**	76	70.5	5 × 10^5^	500 – 1200	2.8	7
**8**	**MEL08**	34.1	60.9	3 × 10^5^	500 – 1200	5.3	16
**9**	**MEL09**	95	84	4 × 10^4^	350 – 800	4.5	10
**10**	**MEL10**	78.7	62.5	2 × 10^5^	600 – 1200	4.2	16
**11**	**MEL11**	77.3	9.56	3 × 10^5^	400 – 1000	2.7	3
**12**	**MEL12**	34.3	15.4	6 × 10^4^	400 – 1200	3.9	10
**13**	**MEL13**	72	9.14	2 × 10^5^	750 – 2000	4.4	16
**14**	**MEL14**	NA	13.2	1 × 10^5^	400 – 10000	1.8	4
**15**	**MEL15**	60	41.5	3 × 10^5^	500 – 4000	4.1	8

**Average**		57.8	38.6	2 × 10^5^	450 – 3250	3.7	10

Whereas the SMART method was reported to maintain the relative levels of RNAs contained in the original transcriptome regardless of their size or their baseline expression [32], the cloning step in *E. coli *was on the contrary described to introduce a bias favoring short fragments [33]. We thus analyzed the quality of the produced amplified-mRNA libraries to be used as a vaccine in melanoma patients. Several clones randomly picked-up within the produced libraries were analyzed by PCR and sequenced. In addition, the gene expression profiles of two metastases were compared to their corresponding cRNA-libraries.

## Results and discussion

### Tumor-derived mRNA library quality

**Figure 1:**
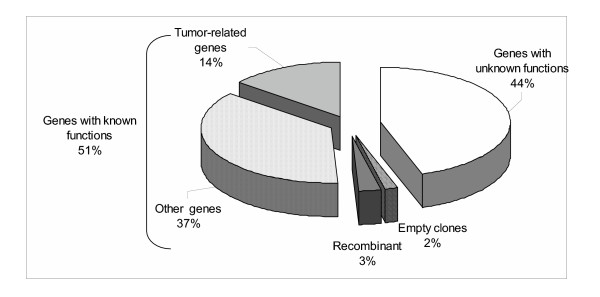
**BLAST analysis of sequenced clones**. Nine clones per library were randomly picked up and their plasmid DNA was sequenced using a T7 promoter primer. Readable sequences (n = 112) were submitted to a BLAST analysis and their relative distribution plotted.

Total RNA was extracted form 15 fresh melanoma tissues. It was then used to generate cDNA libraries according to the SMART protocol (BD Clontech, Palo Alto, CA). The obtained cDNA libraries were ligated into the RNActive™ vector (CureVac GmbH, Tübingen, Germany) containing the mRNA stabilizing sequences of 5' and 3' UnTranslated Regions (UTR) from β- and α-globin respectively [34,35]. Moreover, this vector introduced a 70 A poly(A) tail further enhancing mRNA translation potential (data not shown). RNActive™ libraries were transformed into ultracompetent *E. coli*. The total primary transformant numbers were ranging from 6 × 10^4 ^to 5 × 10^5 ^clones with an average number of 2 × 10^5 ^(table 1). In order to determine the ligation efficiency, 24 clones per library were randomly picked up and submitted to 35 PCR cycles using primers flanking the cDNA insertion sites. Ninety-eight percent of the 312 analyzed clones had an insert with sizes ranging from 200 bp to 10 kbp (data not shown). In order to further test the quality of the libraries, plasmid DNA of 9 clones randomly picked up were extracted for 5'end sequencing (Figure 1). Out of the 112 readable sequences, 2 clones had no insert confirming the 2% rate of self-ligation found by PCR analysis. Among the remaining 110 clones, 3 (3%) showed sequences classified as aberrant with insert sizes inferior to 50 bp probably corresponding to recombination events. The other 107 sequences were analyzed BLAST [36] (Basic Local Alignment Search Tool) using the nr database. About half of the clones (51%) corresponded to full Open Reading Frames (ORFs) of annotated sequences. The other sequences were homologous to ESTs (Expressed Sequence Tags) coding for potential proteins with unknown functions. Full-length clone sizes ranged from 344 to 5925 bp with an average size of 1395 bp correlating with the average insert size of 1.4 kb in cDNA libraries described by Draper *et al*. [37]. Interestingly, 28 % of the clones which aligned to annotated ORFs (14% of all sequenced clones) were tumor related genes (for instance S100 Calcium binding protein A4-metastasin [38]), or genes reported to be overexpressed in tumors (for instance Laminin receptor [39]). This observation fitted with the objective of using the cRNA libraries as anti-tumor vaccines.

### Relative representation of transcripts

**Figure 2:**
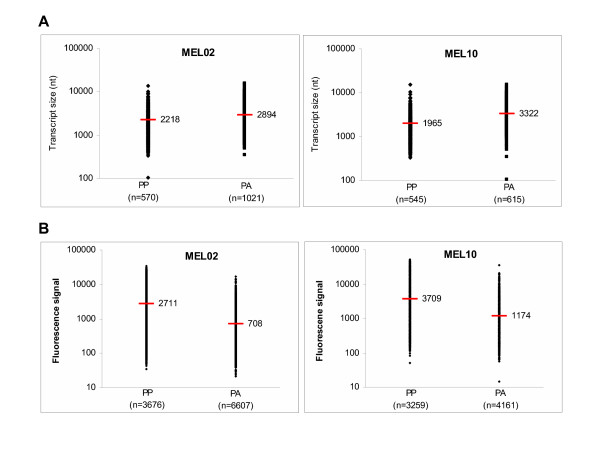
**Comparison of sizes and fluorescence signal intensities in the original tumor sample for "lost" and "recovered" transcripts**. A. The sizes of transcripts Present in tumor's total RNA and reported as Present (PP) or Absent (PA) in the cRNA library of MEL02 and MEL10 patients were plotted. The average size of "recovered" genes was significantly lower (*t test, P < 0.0001*) than the average size of "lost" genes. B. The fluorescence signals of genes "Present" in the original tumor were compared for the transcripts reported as Present (PP) or Absent (PA) in the corresponding mRNA libraries. The group of "recovered" genes showed a significantly higher (*t test, P < 0.0001*) mean signal than the group of genes "lost" during the library production.

In order to determine whether a bias was introduced by the amplification protocol, the relative gene expression in extracted total RNA from two metastases was compared to the relative gene expression in the corresponding amplified cRNA libraries. Biotin-labeled complementary RNA of tumor total RNA and of amplified cRNA libraries from patients MEL02 and MEL10 were generated using the Affymetrix eukaryotic sample and array processing standard procedure and hybridized on HG-U133A microarrays. According to the Microarray Analysis Suite 5.0 software (MAS 5.0; Affymetrix), 34% and 36% of the genes that were reported as "present" in the tumor total RNA were also detected as "present" in the amplified libraries of patients MEL02 and MEL10 respectively (Theses transcripts are qualified as "recovered" in the following). The other transcripts reported by the Microarray Analysis Suite 5.0 software as "present" in tumor's mRNA but as "absent" in the corresponding cRNA library were termed as "lost". In order to determine the factors influencing the biased amplification of genes, the size distributions of "lost" and "recovered" transcripts were compared and plotted in figure 2A. For both patients MEL02 and MEL10, the average size distribution of "recovered" genes (2218 and 1965 nt respectively) was significantly lower (*t test, P < 0.0001*) than the average size of transcripts lost during the amplification process (2894 and 3222 nt respectively). This suggests a biased amplification disfavoring large fragments as observed by Wellenreuther *et al*. [33]. In addition, the fluorescence values in the original tumor of genes reported as "recovered" and "lost" in the cRNA were compared as shown in figure 2B. Average signals of 2711 and 3709 for MEL02 and MEL10 respectively were observed for the genes present in the cRNA library and thus preserved during the process. In contrast, the genes "lost" during amplification had an average signal of only of 708 and 1174 for patients MEL02 and MEL10 respectively. These values were significantly lower (*t test, P < 0.0001*) than those found for the group of "recovered" genes. Thus, transcripts of higher abundance in the original tumor were preferentially preserved during the amplification process whereas mRNAs of lower abundance were eventually lost.

**Figure 3:**
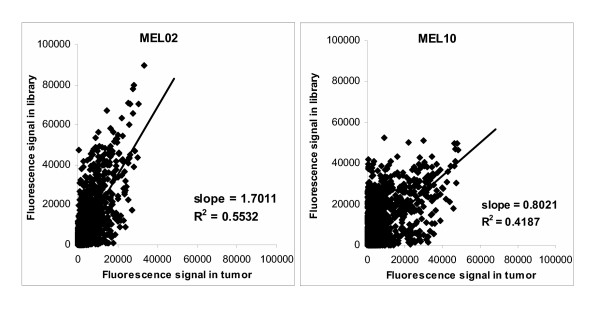
**Correlation of signal intensities in tumor and corresponding mRNA libraries for patients MEL02 and MEL10**. Fluorescence signals in original metastases and amplified tumor cRNA libraries for patients MEL02 and MEL10 were compared for all genes reported as "Present" in the library by MAS 5.0 software.

The fluorescence signal intensities of the genes reported as "present" in both the tumor and the corresponding cRNA libraries were plotted in figure 3. The correlation factors to a linear regression were 0.55 and 0.42 for patients MEL02 and MEL10 libraries respectively, confirming that the mRNA amplification was quite heterogeneous. However, in the group of "recovered" transcripts no significant correlation was evidenced between the transcript size or their signal intensity in the tumor of origin and their amplification factor during the cloning (data not shown).

### Patient-specific gene expression

**Figure 4:**
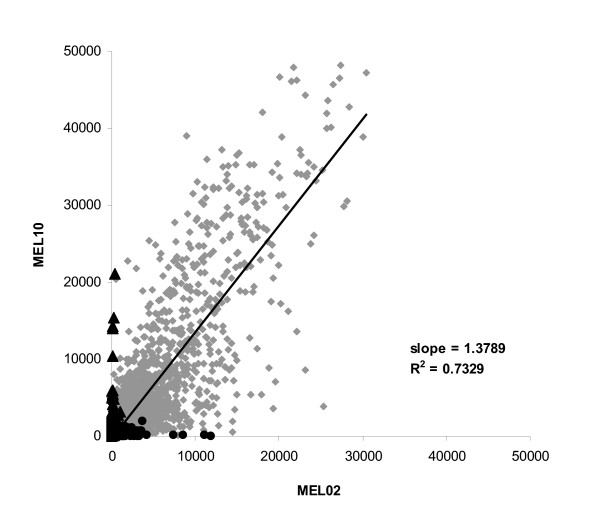
**Correlation of fluorescence of genes present in tumors of patients MEL02 and MEL10**. Fluorescence signals of patients MEL02 and MEL10 tumor transcripts reported by MAS 5.0 software as present in both samples (6993 genes, ◆), only in MEL02 metastasis (3222 genes, ●) or only in MEL10 melanoma (483 genes, ▲).

In order to evaluate the relevance of the autologous approach, signal intensities for the genes present in metastases of MEL02 and MEL10 were compared in figure 4. As expected, the two melanoma samples were quite similar with a correlation coefficient to a linear regression of 0.75 for the 6693 genes shared between the two tumors. However, the two tissues showed specific profiles with 3222 and 483 mRNA transcripts expressed only in patient's MEL02 and MEL10 metastasis respectively. These patient-specific antigens might represent particularly interesting immunological targets [13] and argue for the injection of autologous tumor cRNA rather than the use of cRNA library derived from tumor cell lines as a vaccine.

### Several tumor antigens are present in the cRNA libraries

The transcripts showing the highest fluorescence signals in the injected cRNA library were listed in Table 2 for patient MEL02. Among the 30 genes displaying the highest signals likely representing most abundant transcripts, 13 have already been described as being involved in tumor genesis or observed as being overexpressed in different cancer types. The well-defined and widely used tumor antigen Melan-A was found as one of the most abundant transcripts showing the relevance of cRNA libraries as melanoma vaccines. In addition, the presence in the injected library of many other "tumor-related" antigens rarely used in vaccines targets highlights the potential of such a product to vaccinate patients against a large panel of TAAs.

**Table 2:** List of the thirty transcripts showing the highest fluorescence signals in MEL02 amplified cRNA library

	**Title**	**Symbol**	**Observations**
1	Ribosomal protein L23a	RPL23a	
2	Eukaryotic translation elongation factor 1 alpha 1	EEF1A1	
3	Ribosomal protein S3A	RPS3A	
4	RNase A family, 1 (pancreatic)	RNASE1	
5	Peptidylprolyl isomerase A, cyclophilin A	PPIA	Overexpressed in several cancers [42]
6	Ribosomal protein S23	RPS23	
7	Ribosomal protein L39	RPL39	
8	Melan-A	MLANA	Melanoma differentiation antigen [2]
9	Ribosomal protein L31	RPL31	
10	Cytochrome c oxidase subunit VIc	COX6C	Overexpressed in carcinomas [43]
11	Ribosomal protein L7	RPL7	Overexpressed in gliomas [44]
12	Ribosomal protein L37a	RPL37A	
13	Ribosomal protein S29	RPS29	
14	Secreted phosphoprotein 1, osteopontin	SPP1	Important for tumorgenesis [45]
15	Calmodulin 2	CALM2	Overexpressed in several cancers [42]
16	Ribosomal protein S11	RPS11	
17	"Ribosomal protein S4, X-linked"	RPS4X	
18	Nascent-polypeptide-associated complex alpha	NACA	Overexpressed in gliomas [44]
19	Ribosomal protein L23a	RPL23A	Involved in tumor proliferation [46]
20	ATP synthase, mitochondrial F0 complex, subunit g	ATP5L	
21	Tubulin, alpha, ubiquitous	K-ALPHA-1	Overexpressed in breast cancers [47]
22	NADH dehydrogenase (ubiquinone) 1 alpha subcomplex	NDUFA4	
23	Ribosomal protein S27a	RPS27A	Overexpressed in breast cancers [48]
24	Beta-2-microglobulin	B2M	
25	H2A histone family, member Z	H2AFZ	Overexpressed in several cancers [42]
26	SRY (sex determining region Y)-box 4	SOX4	Overexpressed in lung cancers [49]
27	ATP synthase, mitochondrial F1 complex, epsilon subunit	ATP5E	
28	Tumor protein, translationally-controlled 1	TPT1	Involved in malignant transformation [50]
29	Cytochrome c oxidase subunit VIIa polypeptide 2	COX7A2	
30	Ubiquitin B	UBB	Related to sustained proliferation [51]

## Conclusion

In order to vaccinate metastatic melanoma patients with autologous amplified tumor-derived cRNA, fifteen libraries were produced using a modified SMART method. Despite a heterogeneous amplification of tumor genes, this method provided us with an unlimited source of tumor and patient specific TAAs. Indeed, the microarray analysis of the libraries indicated the presence of high copy numbers of well-known tumor associated antigens such as Melan-A but also of abundant tumor-related antigens scarcely targeted in immunotherapy. Although not addressed in the present work, this method might also allow the targeting of tumor-specific mutations. These features makes of the amplification of tumor mRNA the method of choice to easily obtain unlimited amounts of RNA coding for patient's specific TAAs that can be applied as anti-tumor immunotherapy.

## Materials and methods

### Tumor samples

Immediately after surgery, metastatic tissues from fully informed patients (Ethic committee approval Nr.: 269/2002) were chopped in ~0,1 cm^3 ^pieces, and submerged in RNAlater solution from Ambion (Hungtingdon, UK), and stored at 4°C until histological identification as melanoma by an experienced pathologist.

### Tumor total RNA extraction

Total RNA was extracted from tumors using the RNeasy mini kit from Qiagen (Hilden, Germany) following the instructions of the provider. Briefly, 15 to 30 mg samples placed in a 2 ml eppendorf tube were snap-frozen in a liquid nitrogen bath and disrupted with micropistils from Eppendorf (Hamburg, Germany). Tumor powder was resuspended in RLT buffer, homogenized through a 20-gauge needle and digested with 200 μg of proteinase K (Qiagen) at 55°C during 10 min. Samples were then clarified, loaded on RNeasy mini columns, washed and finally eluted in 50 μl of RNAse-free water. RNA was quantified by U.V spectrophotometry (O.D_260_/O.D_280 _ratio was over 1.8 in all cases) and analyzed on a 1,2% formaldehyde/agarose gel.

### cDNA library generation

cDNA libraries of tumor total RNA were generated using the slightly modified Creator™ SMART™ PCR cDNA library construction kit from BD Biosciences Clontech (Heidelberg, Germany). Briefly, 1 μg of total RNA was reverse transcribed using SMART IV™ and CDS III/3' oligo-dT primers provided by the manufacturer. After termination of the reaction, 2 μl of cDNA were amplified using the Advantage 2 PCR kit (BD Biosciences Clontech). DNA polymerase was then inactivated with proteinase K and the cDNA library was digested with 200 U of *Sfi *I enzyme. cDNA libraries were then gel-purified on an 1% agarose gel and fragments from 300 bp to 10 kbp were extracted using E.Z.N.A.™ Gel Extraction kit from Peqlab GmbH (Erlangen, Germany). After precipitation, the cDNA library was ligated to dephosphorylated *Sfi *I-digested RNactive™ vector provided by CureVac GmbH (Tübingen, Germany) in three separated reactions to optimize vector/insert ratios.

### Cloning

The three ligation products were used to transform XL10-Gold ultracompetent cells from Stratagene (Heidelberg, Germany). For analysis, 1 and 10 μl of transformation broth were plated on 2 LB-ampicillin agar plates and, after overnight culture at 37°C, the number of clones was counted. The inserts of 8 clones per transformation were amplified by PCR using primers flanking the insertion sites and amplicons were analyzed on a 1% agarose gel. Libraries having more than 10^4 ^clones/ml and less than 20% of non recombinant clones, were amplified in three 300 ml maxicultures in 2X LB-ampicillin medium during 20 h at 33°C in order to limit uneven amplification of clones.

### DNA preparation and linearization

Maxicultures were pooled, centrifuged down at 5 000 rpm for 10 min and plasmid DNA was extracted using EndoFree Plasmid Maxi (Qiagen). After precipitation, 100 μg of cDNA library were digested with 100 U of *Not *I enzyme. After phenol/chloroform extraction and ammoniumacetate precipitation, linearized cDNA libraries were resuspended in RNAse-free water, quantified by U.V. spectrophotometry (O.D_260_/O.D_280 _ratio was over 1.8 in all cases) and analyzed on 1% agarose gel.

### cRNA *In vitro *transcription

Twenty to hundred micrograms of linear cDNA library were *in vitro *transcribed using T7 mRNA Optikit from CureVac GmbH. After mRNA synthesis, DNA template was digested with 40 to 100 U of recombinant DNAse I purchased from Ambion. mRNA was then LiCl precipitated, phenol/chloroform purified, NaCl precipitated, and finally resuspended in PBS. cRNA was filter sterilized (0,2 μm), heat denatured at 80°C for 10 min before final sterile aliquoting. cRNA was quantified by U.V spectrophotometry (O.D_260_/O.D_280 _ratio was over 1.8 in all cases) and analyzed on 1.2% formaldehyde/agarose gel. Sterility of cRNA was checked by inoculating LB medium (in all cases, no bacterial growth was observed after 4 days at 37°C) and endotoxin content was determined using Bio-Whittaker (Verviers, Belgium) LAL assay kit (endotoxin content was always below 7 EU/ml).

### Clone sequencing

For each library, 3 colonies per transformation were randomly picked-up with a pipette tip and used to inoculate 3 ml of LB-ampicillin medium. After overnight culture at 37°C, plasmid DNA was extracted using E.Z.N.A miniprep kit (Peqlab). Clone sequencing was performed using the ABI Big Dye and a T7 promoter primer. Sequences were purified on Autoseq. G-50 columns (Amersham Pharmacia Biotech, Freiburg, Germany), run on a 310 Genetic Analyzer from ABI PRISM™ (Applied Biosystems, Darmstadt, Germany) and analyzed with the Sequencing Analyzing 3.4.1 software (ABI PRISM). Finally, the BLAST algorithm [36] was used to identify matches to known genes.

### Microarray analysis

Expression analysis of total tumor RNA and amplified tumor cRNA was performed on HG-U133A microarrays from Affymetrix (High Wycombe, UK) according to the manufacturer's eukaryotic sample and array processing standard procedure [40], which is based on the IVT method originally described by Van Gelder *et al*. [41]. Briefly, 1^st^-strand cDNA synthesis was performed using an oligo(dT)24 primer containing a T7 promoter sequence. After RNA template degradation and cDNA's second strand cDNA synthesis, complementary RNA (cRNA) was transcribed *in vitro *using biotinylated NTPs and T7 RNA polymerase. After purification using RNeasy columns (Qiagen), 18 μg of biotin-labeled cRNAs were fragmented by metal-induced hydrolysis. Hybridization, staining, and scanning of microarrays were performed by the Microarray Facility Tübingen. Scanned images were processed using the Microarray Analysis Suite 5.0 (MAS 5.0; Affymetrix) and expression differences between tumor and library samples were determined by baseline comparison algorithms provided by the software. Data were further processed using Microsoft Access™ and Excel™.

## Authors' contributions

JPC carried out the total tumor RNA extraction, the production of mRNA libraries, the analysis of clones, participated in the microarray analysis of RNA samples, evaluated the microarray data and drafted the manuscript. OS carried out the microarray analysis of RNA samples with the help of the Microarray Facility Tübingen. BW and CG carried out the patient's recruitment and the tumor sample preparation. JP, BS and RT participated in the design of the study, the development of the work and helped to draft the manuscript. CG, IH, HGR and SP conceived, designed and coordinated this study. All authors read and approved the final manuscript.
